# Routes of Administration of Illicit Drugs among Young Swiss Men: Their Prevalence and Associated Socio-Demographic Characteristics and Adverse Outcomes

**DOI:** 10.3390/ijerph182111158

**Published:** 2021-10-24

**Authors:** Natalia Estévez-Lamorte, Simon Foster, Gerhard Gmel, Meichun Mohler-Kuo

**Affiliations:** 1 Department of Child and Adolescent Psychiatry and Psychotherapy, University Hospital of Psychiatry Zurich, University of Zurich, 8032 Zurich, Switzerland; simon.foster@uzh.ch (S.F.); m.mohler-kuo@ecolelasource.ch (M.M.-K.); 2 Alcohol Treatment Centre, Lausanne University Hospital CHUV, 1011 Lausanne, Switzerland; Gerhard.Gmel@chuv.ch; 3 Addiction Switzerland, 1001 Lausanne, Switzerland; 4 Centre for Addiction and Mental Health, Toronto, ON M5S 2S1, Canada; 5 Department of Health and Social Sciences, University of the West of England, Bristol BS16 1QY, UK; 6 La Source, School of Nursing Sciences, HES-SO University of Applied Sciences and Arts of Western Switzerland, 1004 Lausanne, Switzerland

**Keywords:** routes of drug administration, oral use, nasal use, smoking, injecting, alcohol use disorder, suicidal ideations, social consequences, health consequences

## Abstract

The prevalence of different routes of administration (ROAs) of illicit drugs other than cannabis was examined in young Swiss men, in addition to the association between socio-demographics and adverse outcomes and particular ROAs. Our sample consisted of 754 men (mean age = 25.4 ± 1.2 years) who participated in the Cohort Study on Substance Use Risk Factors and reported using any of 18 illicit drugs over the last 12 months. Prevalence estimates were calculated for oral use, nasal use, smoking, injecting, and other ROAs. Associations between ROAs and socio-demographics and adverse outcomes (i.e., alcohol use disorder (AUD), suicidal ideations, and health and social consequences) were calculated for using single versus multiple ROAs. The most prevalent ROA was oral use (71.8%), followed by nasal use (59.2%), smoking (22.1%), injecting (1.1%), and other ROAs (1.7%). Subjects’ education, financial autonomy, and civil status were associated with specific ROAs. Smoking was associated with suicidal ideations and adverse health consequences and multiple ROAs with AUD, suicidal ideations, and health and social consequences. The most problematic pattern of drug use among young adults appears to be using multiple ROAs, followed by smoking. Strategies to prevent and reduce the use of such practices are needed to avoid adverse outcomes at this young age.

## 1. Introduction

Illicit drugs can be used through different routes of administration (ROAs), such as oral use, nasal use, smoking, and injecting. The way in which drugs are taken affects how effectively and rapidly they are absorbed and metabolised by the body and, consequently, influences the risk of developing dependence and the types of harm experienced by drug users [1,2].

Despite having important implications on users’ health outcomes, ROAs are not well studied. Most previous research has focused on injected drug use and related consequences, because this is considered the most harmful route of drug administration and is often used by consumers with more severe use patterns (e.g., see [1,3,4,5,6]). Users who consumed drugs through other routes were, when included, often coalesced into a single group (i.e., non-injectors), and used as a comparison group in studies exploring injection as the main ROA. However, even though these other ROAs appear to be less dangerous than injection drug use, they are far from harmless [7,8]. The consumption of drugs by such methods is associated with considerable health and social consequences, including death [9,10,11,12,13]. In addition, using illicit drugs through less harmful ROAs can transition to more risky methods of use [1,14,15], thereby increasing the risk of developing more serious adverse outcomes [7,8,15]. Furthermore, the lack of distinction between various ROAs, when treated as a single group, can mask important differences between people who consume their drugs through different ROAs, knowledge of which might aid in the development and administration of interventions tailored to individual users (e.g., addressing route-specific consequences) [10]. Finally, the use of particular ROAs can vary over time [16,17,18]. Although changes are difficult to predict, regular assessments of current ROAs can help to monitor how drugs are taken and provide up-to-date information needed to develop appropriate prevention programs. For all these reasons, prevalence studies on the ROAs of illicit drugs that take different methods of use into account are crucial to enabling the development of preventive strategies and interventions that best address current trends in drug use and the actual needs of drug users.

Previous investigations assessing prevalence rates for various ROAs (i.e., other than injection vs. non-injection) have often used convenience samples drawn from high-risk populations (e.g., individuals recruited from treatment settings [18,19], captured individuals [20,21], regular users of specific substances [12], and emergency departments [9,13]), which may consist mainly of users with more severe patterns of use (e.g., individuals who inject or are addicted). Consumers of illicit drugs with no contact with health or legal services often were not included (for review, see [1]). Thus, little is known about their preferred ROAs or associated socio-demographic characteristics or the harms that they may experience related to particular ROAs. Studies using more heterogeneous samples drawn from the general population may provide a more comprehensive picture of how illicit drugs are currently taken. 

With respect to ROAs of illicit drugs, young adulthood is of particular interest because it is during this phase of life that individuals, men especially, start to experiment with illicit drugs other than cannabis [22]. In addition, it is during this phase of life that individuals must face a number of normative development tasks, which involve substantial changes in every life domain [23]. Developing drug addiction or experiencing drug-related adverse social and health outcomes (e.g., having problems with the police) during this critical stage may make it more difficult for individuals to deal with the challenges that this phase introduces (e.g., initiating professional life, finding a job), causing long-term negative effects that persist into later adult life. Acquiring more thorough knowledge about the prevalence of the ROAs of illicit drugs, their associated socio-demographic factors, and related harms in young adults could aid in identifying vulnerable groups, which, in turn, could help to prevent negative consequences later in life.

In Switzerland, few studies have addressed the prevalence of the various ROAs of illicit drugs [9,13,16,21,24]. Recent prevalence estimates of ROAs among young men drawn from the general population stem from a single study. However, this investigation focused on the illegal use of cannabis only [24]. Thus, to our knowledge, no recent study has addressed methods of use of other illicit drugs. For this population, also, no data exist on socio-demographic factors and harms associated with the administration of drugs through particular routes. 

Therefore, the aims of the present study were (1) to assess the prevalence of ROAs of illicit drugs other than cannabis within a sample of young Swiss men; (2) to identify socio-demographic factors associated with the ROAs most commonly used by this population; and (3) to explore the relationship between the ROA used and the presence of particular adverse outcomes in young men. For the present study, we used data from an ongoing cohort study (‘Cohort Study on Substance Use Risk Factors’, C-SURF) that has been collecting data from the general population to investigate substance use patterns among young Swiss men over time. To meet the second and third aim of this investigation, we focused on ROAs that were commonly used by our sample. This included oral use, nasal use, and smoking. We differentiated between subjects who consumed illicit drugs exclusively by one of these methods from those who used multiple ROAs. This approach was used to avoid confounding effects between ROAs (i.e., to ensure that observed associations are related to a particular ROA), and to allow us to investigate whether using multiple ROAs differs from using a single ROA, in terms of other socio-demographic characteristics and adverse outcomes, an issue that has only been explored superficially [11,12]. With respect to adverse outcomes, we focused on outcomes assessed in C-SURF and previously reported among illicit drug users, including mental health problems, such as alcohol use disorder (AUD) and suicidal ideations, and social and other health (medical) consequences [10,11,12,25].

## 2. Materials and Methods

### 2.1. Study Design

Data were drawn from C-SURF, a longitudinal study designed to investigate substance use patterns within a cohort of young Swiss men. The C-SURF protocol was approved by the Ethics Committee for Clinical Research at Lausanne University (protocol number 15/07). Informed written consent was obtained from all participants prior to data collection.

Participants were recruited at three of the six centres that recruit men for military service in Switzerland, covering 21 of the 26 Swiss cantons, including all French-speaking and most of the German-speaking regions. In Switzerland, army recruitment is mandatory; thus, all Swiss men must attend a formal evaluation to determine their eligibility for military service, civil service, or no service at around age 19. Both those who were deemed eligible for military or civil service and those deemed ineligible to serve in the army were eligible for enrolment in our study. As there is no pre-selection to army conscription, this procedure provided us with access to a representative sample of young Swiss men for the 21 cantons covered by the participating recruitment centres. Note that these army centres were used exclusively to enrol the participants. All other aspects of the study and subject participation were totally independent of the army. Participants were first enrolled for the study between August 2010 and November 2011. To date, data have been collected in four waves (a baseline assessment and first, second, and third follow-up assessments). All the data on the ROAs of illicit drugs were collected during the second follow-up, between April 2016 and March 2018, when the mean age for all participants was 25.5 ± 1.3 years.

### 2.2. Participants

From the 7563 men who initially gave written consent to participate, 5516 (72.3%) completed the second follow-up questionnaire. Of these, 754 (13.7%) reported having used illicit drugs other than cannabis over the preceding 12 months. In the current study, prevalence estimates for ROAs are reported for this last group of 754 participants. For further analyses (i.e., in multinomial logistic regression and binomial logistic regression analyses), an additional 43 individuals were excluded. This included participants who reported using ROAs not included in multivariable analyses (e.g., injecting) or who had data missing on socio-demographic variables. Thus, for multivariable analyses, the final sample consisted of 711 subjects. 

### 2.3. Measures

#### 2.3.1. Routes of Administration 

Routes of administration (ROAs) were assessed for 18 different illicit drugs, including (1) natural hallucinogens (magic mushrooms, psilocybin, peyote, mescaline); (2) other synthetic hallucinogens (lysergide (LSD), phencyclidine (PCP, angel dust), 4-bromo-2,5-dimethoxyphenethylamine (2-CB), 2,5-dimethoxy-4-iodophenethylamine (2-CI)); (3) salvia divinorum; (4) amphetamine/speed, amphetamine sulphate (e.g., dexedrine, benzedrine); (5) khat; (6) methamphetamine (Thai pills, crystal meth (ice)); (7) poppers (amyl nitrite, butyl nitrite); (8) solvents for inhalation (e.g., glue, other solvents, and gases such as benzine, ether, toluol, trichloroethylene, nitrous oxide); (9) ecstasy, 3,4-methylenedioxy-N-methylamphetamine (MDMA); (10) cocaine, crack, freebase; (11) heroin, morphine, opium; (12) ketamine (special K), dextromethorphan (DXM (Bexin)); (13) methadone; (14) GHB/GBL/1,4 butandiol (BDO); (15) bath salts, research chemicals, other legal highs (e.g., MDPV, mephedrone, butylone, and methedrone); (16) spices or similar substances to smoke that can contain synthetic cannabinoids; (17) Ayahuasca/DMT, psychedelic rainforest plants; and (18) ibogaine. 

Participants were asked to indicate whether or not they had used each of the afore-mentioned substances within the last 12 months. For each drug, answers were coded as ‘use’ or ‘no use’. Those who reported using illicit drugs were then asked how each of the substances that they used was administered. Five ROAs were considered: oral use (i.e., oral ingestion of tablets, capsules, fluids, food (e.g., cookies)), nasal use (i.e., sniffing, snorting), smoking or inhaling, and injecting (i.e., intravenous, intramuscular, or subcutaneous injection or injecting into the bone), as well as an unspecified category called ‘other ROA’ (e.g., chewed, transcutaneous, rectal). Multiple responses were possible. Accordingly, answers were coded as ‘oral use’, ‘nasal use’, ‘smoking’, ‘injecting’, and ‘other ROA’. This information was provided for each of the 18 illicit drugs separately, and for using any of these drugs. 

For analyses examining the most commonly used ROAs, an additional variable was created. This differentiated between the use of single and multiple ROAs and included four categories: (1) ‘oral use only’; (2) ‘nasal use only’; (3) ‘smoking only’; and (4) ‘use of multiple ROAs’.

#### 2.3.2. Socio-Demographic Variables 

Socio-demographic variables included the subject’s current age (‘younger than 25′ vs. ‘25 years or older’), linguistic region (‘German-’ vs. ‘French-speaking’), education level (‘primary/secondary school’ vs. ‘high school/university’), degree of financial autonomy (‘financial autonomy’ vs. ‘partial financial dependency’ vs. ‘financial dependency’), and civil status (‘in a relationship’ vs. ‘single’).

#### 2.3.3. Adverse Outcomes 

##### Alcohol Use Disorder (AUD)

Symptoms of AUD within the past twelve months were assessed using a questionnaire [26] adapted from the Semi-Structured Assessment for the Genetics of Alcoholism (SSAGA) [27,28]. In accordance with the DSM-5 criteria [29], the ‘likely presence’ of AUD was defined as a positive response to at least two of eleven symptoms, and coded as a binary variable (‘no AUD’ vs. ‘AUD’).

##### Suicidal Ideations

The presence of suicidal ideations was assessed using a single question from the Self-Injurious Thoughts and Behaviours Interview (SITBI; [30]). Participants were asked whether or not they had ever had thoughts of killing themselves. Answers were coded as (‘no suicidal ideations’ vs. ‘suicidal ideations’).

##### Social and Health Consequences

Participants were asked whether they had or had not experienced any of 15 drug-use-related consequences within the last 12 months [27,28,31,32]. These items included social and physical health (medical) consequences. For each consequence, answers were coded as ‘no’ if it had not been experienced or ‘yes’ if it had taken place at least once in the past 12 months. Social consequences included physical fights, problems with parents/family, problems with friends, poor performance at school/work, theft, problems with the police, regretted sexual intercourse, and damage to property. Health consequences included accident/injury, admittance to an emergency department, need for medical treatment, overnight stay in a hospital, outpatient surgery, treatment of an accident/injury in an emergency department, and treatment at a specialised centre for problems of dependence to toxic substances. Except for the last consequence, these items were assessed without mentioning an explicit substance involvement.

### 2.4. Statistical Analysis

Data were analysed using the statistical package SPSS 27.0 (IBM Corp., Armonk, NY, USA). Contingency tables were used to present prevalence rates for ROAs among users of any of the 18 illicit drugs and for each of these drugs separately. Prevalence estimates also were calculated for the use of single and multiple ROAs among users of any of the illicit drugs. Socio-demographic and adverse outcome variables were compared between participants who used any of the illicit drugs of interest only orally, nasally, or by smoking or through multiple ROAs using Pearson chi-square analysis or Fisher’s exact test (whenever expected frequencies were under 5%).

Multinomial logistic regression analyses were conducted to examine the association between the most commonly used ROAs (i.e., oral use only, nasal use only, smoking only, and use of multiple ROAs) among users of any of the illicit drugs and socio-demographic variables entered as potential predictors. Unadjusted and adjusted odd ratios (OR) and 95% confidence intervals (CI) were computed for all predictors. Adjusted values were assessed using all socio-demographic variables as covariates.

Binomial logistic regression analyses were performed to investigate associations between the most commonly used ROAs among users of any of the illicit drugs and adverse outcomes. Odd ratios and CI were calculated, both unadjusted and adjusted for socio-demographic variables, using ROAs of interest as predictors and AUD, suicidal ideations, and social and health consequences as outcome variables. For consequences, analyses were performed for each consequence separately.

## 3. Results

### 3.1. Prevalence of ROAs of Illicit Drugs

Prevalence rates for ROAs among users of any illicit drug and for each illicit drug included in the study separately are shown in Table 1. The most prevalent ROA was oral use (71.8%), followed by nasal use (59.2%) and smoking (22.1%), while only a few users reported taking drugs by injection (1.1%) or using other ROAs (1.7%) and 3.3% did not provide information about the ROA used. Nearly half of the illicit drug users (47.7%) reported using more than one ROA. Roughly a quarter of the young men used illicit drugs only orally (26.8%), while 16.2% of them used illicit drugs only nasally, 5.7% only by smoking, and 0.4% only through injecting or other ROAs (for details on use of single and multiple ROAs, see Table 2). Among those conscripts who had used illicit drugs in the past 12 months, the drugs used most commonly were ecstasy (57.7%); cocaine, crack, freebase (47.7%); amphetamine/speed, amphetamine sulphate (30.2%); natural hallucinogens (23.3%); poppers (19.8%); and other synthetic hallucinogens (19.0%). Conscripts reported having used these drugs through oral digestion, nasally, and/or smoking. Ecstasy, natural hallucinogens, and other synthetic hallucinogens were more often used orally, while nasal use was preferred for cocaine, crack, freebase, amphetamine/speed, amphetamine sulphate, and poppers. For detailed information on the use and administration of each of the 18 illicit drugs, see Table 1.

Given the small number of participants who used drugs by injection and through other ROAs, further analyses focused solely on oral use, nasal use, smoking, and the use of multiple ROAs.

### 3.2. Socio-Demographic Variables

Socio-demographic characteristics of the study sample are summarised in Table 3. Results of the multinomial logistic regression analyses are summarised in Table 4.

Subjects’ education, degree of financial autonomy, and civil status were associated with different ROAs. Compared to participants who used illicit drugs only orally (reference category), conscripts having completed primary or secondary vocational school only were more likely to report nasal use and the use of multiple ROAs than those having a high school or university level of education. After adjusting for socio-demographics, ORs remained significant for the use of multiple ROAs, but not for nasal use.

Compared to conscripts who used drugs only orally (reference category), men who reported financial autonomy were more likely to consume illicit drugs nasally than those who reported partial or complete financial dependency. Similarly, participants reporting financial autonomy were more likely to consume illicit drugs by smoking than those reporting partial financial dependency. After adjustment, the negative relationship between partial financial dependency and smoking remained significant. In contrast, for the negative associations between partial and complete financial dependency and nasal use, only a trend was observed after adjusting for socio-demographics.

Compared to participants who consumed only orally (reference category), conscripts who were in a relationship were more likely to smoke than participants who were single. However, for this association, only a trend was detected when adjusting for socio-demographic variables.

Multinomial logistic regression analyses, both unadjusted and adjusted for socio-demographics, identified no significant link between any of the ROAs included in the analyses and age or linguistic region. Furthermore, no significant associations were found between smoking and education, use of multiple ROAs and financial autonomy, or between nasal use and the use of multiple ROAs and civil status.

### 3.3. Adverse Outcomes

Adverse outcomes by ROA are compared in Table 5. For detailed results of the related binomial logistic regression analyses, see Table 6.

Logistic regression analyses, both unadjusted and adjusted for socio-demographics, showed that the use of multiple ROAs was significantly associated with several adverse outcomes, including AUD, suicidal ideations, experiencing problems with friends, and having an accident/injury or being treated for an accident/injury in an emergency department. In adjusted, but not in unadjusted, analysis, a significant association also was found between using multiple ROAs and regretted sexual intercourse. In this context, men who used multiple ROAs experienced each of the aforementioned outcomes more often than those who ingested drugs only orally. Similar positive associations were observed between the use of multiple ROAs and reporting physical fights, having problems with the police, and being admitted to an emergency department. However, for these relationships, only a trend was observed after adjusting for socio-demographic variables. A trend also was found for the association between the use of multiple ROAs and causing damage to property.

Men who consumed illicit drugs preferably by smoking more often needed medical treatment or treatment for an accident/injury in an emergency department. Adjusting for socio-demographics did not substantially change the results for any of these outcomes. A positive association also was found between smoking and staying overnight in a hospital. However, after adjustment, this association only showed a trend. Furthermore, adjusted logistic regression analyses revealed that participants who used drugs only through smoking were more likely to report suicidal ideations.

With respect to adverse outcomes, participants who used drugs only nasally did not substantially differ from those who preferred oral use.

Moreover, no significant associations were detected between the ROA used and experiencing problems with parents/family, showing poor performance at school/work, being involved in a theft, or having outpatient surgery. Only a few subjects reported being treated at a specialised centre for problems of dependence on toxic substances. Thus, for this variable, the number of cases for each ROA was too small to perform analyses.

## 4. Discussion

In our sample of young Swiss men, we found that illicit drugs were taken preferably through oral ingestion (71.8%), followed by nasal use (59.2%) and smoking (22.1%). Only a few individuals reported having injected their drugs (1.1%) or used other ROAs (1.7%). Drugs consumed orally or nasally are metabolised more extensively by the body, which decreases a drug’s concentration before its final effects become manifest. Smoking and, in particular, injection are more direct methods of use. Drugs consumed by these ROAs are absorbed quickly and delivered in large amounts to the brain, which leads to stronger drug effects and makes these ROAs more dangerous, in terms of developing dependence or experiencing harm [2]. Compared to previously published prevalence reports [19], the young men included in the present study largely seemed to use less risky ROAs. Indeed, in an international report on 24 European countries [19], covering illicit drugs such as cocaine and amphetamines, which were commonly used in our sample and can be consumed via all four ROAs that we examined, the percentages of individuals who reported smoking or injecting these drugs were considerably higher than in our sample (27% smoked and 2% injected cocaine as opposed to 6.4% and 0.8% in our sample; 11% smoked and 9% injected amphetamines as opposed to 1.3% and 0% in our sample). Meanwhile, oral and nasal use were more prevalent among our participants (2% used cocaine orally and 68% nasally, as opposed to 7.8% and 89.4% in our sample; 14% used amphetamines orally and 65% nasally, as opposed to 21.5 and 85.5% in our sample). The discrepancy between these findings can be explained by differences in the populations that these two surveys investigated. In the international report, the prevalence of ROAs was assessed among drug users entering treatment and may, therefore, have included more problematic users than our sample, which, being drawn from the general population, almost certainly encompassed a more heterogeneous group of drug users, including many with less problematic drug use patterns. Such users may consume drugs primarily for experimentation and recreational purposes or to self-medicate an existing mental condition or emotional concerns [34], rather than to alleviate symptoms associated with drug addiction, as may be the case in users with more problematic drug use patterns. Drug users with the former motivations may want to experience the effect of the drug without risking adverse consequences and may, consequently, choose ROAs that are believed to allow more control over the drug and are, therefore, perceived as relatively safe.

In addition, the predominance of oral and nasal administration observed in our sample may be, to some extent, also due to the type of drugs preferred by our young subjects. Ecstasy, natural and other hallucinogens, and poppers, which were among the most commonly used drugs reported by our sample, are mainly available in a form that can be easily taken orally or nasally. Furthermore, the low prevalence rates of injection are most probably due to the rare use of opioids, especially heroin, among our subjects. This drug is considered the main injected drug in the majority of European countries [19]. In Switzerland, its use has declined over the last few decades and is more prevalent among users who are older than our subjects [16,35].

Although our results indicate a preference for less risky over more dangerous ROAs, it must be noted that nearly half of our participants (47.7%) reported using more than one ROA. With our data, we cannot definitively determine whether different ROAs were used on single occasions (e.g., while experimenting with drugs) or whether this practice reflected a more problematic pattern, such as the transition to more risky ROAs or even as a regular use pattern. We observed, however, that the majority of subjects who used more than one ROA (96.7%; data not shown) also reported consuming multiple illicit drugs, while young men who administered their drugs only by one ROA tended to use only one type of drug (79.9%, 86.7%, and 92.9% of consumers who used drugs only orally, nasally, or by smoking, respectively; data not shown). Polydrug use has been associated with more severe patterns of use, including a higher risk of switching to more dangerous ROAs [1,24]. We suspect, therefore, that the participants who reported using multiple ROAs were users with more problematic consumption patterns or at least an elevated risk of developing such patterns. A transition to more risky administration methods or the regular use of more than one ROA may exacerbate the risk of developing dependence and/or increase the number and severity of harms experience by drug users [1,2,12]. In particular, regular use of multiple ROAs could enhance the probability of adverse outcomes. Indeed, it has been shown that drug users tend to be affiliated and share drugs with individuals who use the same types of ROA (e.g., people who smoke, sniff, and inject generally associate with others who smoke, sniff, and/or inject). Consequently, consumers who use multiple ROAs have a larger network of active drug users with whom they share drugs on a regular basis than consumers who use only a single ROA [12]. It is possible that active contact and exchange with a larger network of drug users may increase the risk of an individual developing and/or perpetuating more severe patterns of use (e.g., permanent transition to a more risky ROA) [14], making it more difficult for them to reduce or cease drug use.

### 4.1. Socio-Demographic Factors

We found no significant link between age and ROA. Most previous research addressing this relationship has focused on injected drug use, with most studies specifically investigating age differences between subjects who inject versus those who do not (i.e., coalesced into a single group without discriminating between other ROAs) [4,5,21]. Such investigations have revealed higher rates of injection drug use with increasing age [4,5], suggesting that older users with longer drug histories and more dependence-related symptoms may opt for the ROA that delivers the drug most efficiently to alleviate their symptoms. Given that we did not include injection in our analyses and that we addressed different ROAs to previous studies, our results are not fully comparable with earlier findings. It is, however, possible that our failure to identify any significant relationship between age and ROAs in the present study might also be due to the narrow age range and youth of our subjects. In fact, these previous investigations [4,5,21] included subjects in various age categories (i.e., from adolescence to late adulthood), while our study focused exclusively on young adults (ages 23 to 31 years old). In addition, a significant association with the method of drug use has mainly been observed for subjects who were, on average, older than our study participants (i.e., >31 years old). Thus, the relationship between age and the particular ROAs included in this study needs to be investigated further in studies involving samples spanning a wider age range.

We noted no significant association between linguistic region and ROA. This result stands contrary to findings of previously published studies [7,17,18,19,36] that identified regional variations in ROAs. This inconsistency might be due to differences in sample characteristics. Prior studies investigated relatively homogenous higher-risk populations (e.g., subjects seeking treatment for drug misuse), thereby consisting mainly of subjects with more severe use patterns, while our study collected data from a more heterogeneous population. It is, therefore, possible that, in former investigations, regional variations in ROAs were more salient due to the homogeneity of the samples and the particular characteristics of the subjects under investigation than in the present study. Our sample covered a wider spectrum of users, including those with less problematic patterns (e.g., users who used illicit drugs for experimentation only and first-time users) and provides a better representation of the use of ROAs among the general population of young Swiss men. However, the use of a heterogeneous sample may have prevented us from detecting more subtle differences between ROAs. Nevertheless, we cannot exclude the possibility that young men in the French- and German-speaking areas of Switzerland do, in fact, use similar practices to administer illicit drugs. The way in which drugs are used in a particular region or area may depend on several factors, including social and economic aspects [7,18,21]. Switzerland is a stable country with similar social structures and medical services and no major economic or marginal differences across regions. Under these circumstances, it is possible that, at least with respect to linguistic region and for this particular population, there might be no regional differences in ROAs.

Men having completed primary or secondary vocational school were more likely to report using drugs through multiple ROAs, rather than orally, than those with a high school or university level of education. A significant association between education level and ROAs has been reported before [4,12]. Although these studies investigated other ROAs (i.e., injecting versus not injecting drugs) besides those included in the present study, they also revealed a direct link between lower education level and more dangerous methods of drug administration (e.g., injecting). Our observation that young men using multiple ROAs may be users with more severe consumption patterns, together with these previous findings, indicates that a person’s education level could help to predict their use of more harmful administration practices (e.g., use of multiple ROAs or injected drug use). This particular characteristic, therefore, seems to be a key target for prevention efforts.

Young men who reported being financially autonomous were more likely to prefer smoking over using drugs orally than participants reporting partial financial dependency. Moreover, subjects reporting financial autonomy were more likely to preferentially use drugs nasally over orally than men reporting partial and complete financial dependency. However, for this association, only a trend remained after adjusting for all other socio-demographic variables. The tendency of men who are financially dependent (either partially or completely) to use illicit drugs orally rather than by sniffing or smoking seems to reflect differences in the price of the drugs used. In fact, in a global drug survey [37] spanning more than 25 countries, natural hallucinogens (i.e., magic mushrooms) and synthetic drugs, such as ecstasy, and, in particular, synthetic hallucinogens (i.e., LSD), were rated as providing good value for money, with LSD even being rated as the cheapest drug. In our sample, these drugs were among the most frequently used and mainly were administered orally. In contrast, illicit drugs, which, according to this survey, were more expensive, such as cocaine or solvent sniffing (e.g., nitrous oxide), were more often consumed using other ROAs. For young adults who have not yet achieved financial independence, drug use costs may be of particular importance. In this context, one must consider whether regulations that provide more control over pricing may help to prevent or reduce the use of such drugs, especially among young men [37].

We found a trend between civil status and ROA. In this context, men who were in a relationship were more likely to smoke (versus consume drugs orally) than subjects who were single. Given that smoking may be a riskier ROA, in terms of developing dependence and experiencing adverse consequences, than consuming drugs orally, our findings suggest that being in a relationship could be considered a risk factor. Our definition of a relationship may have contributed to this finding. This variable was defined as being married or living with a partner, and not simply having a girlfriend. It is, therefore, possible that those men who were in a committed relationship were also financially in a more stable situation and could afford more expensive drugs, which, in our sample, were preferably consumed by smoking. Indeed, two thirds of men (50 out of 75; data not shown) who reported being in a relationship were also financially autonomous. Given that, to our knowledge, there are currently no published studies addressing the link between civil status and the ROAs included in the current study, and that we only identified a trend for this association, further research is clearly needed to corroborate and potentially explain this finding.

### 4.2. Adverse Outcomes

Compared to the oral use of illicit drugs, we found that smoking and using multiple ROAs were associated with several adverse outcomes, while individuals who used drugs nasally did not significantly differ from those who used illicit drugs orally. Smoking was linked to suicidal ideations and adverse health consequences, such as needing medical treatment and being treated for an accident or injury in an emergency department. The use of multiple ROAs was associated with even more adverse outcomes, which consisted of mental health problems (i.e., AUD and suicidal ideations), as well as both physical health and social consequences. Health consequences included having an accident or injury and being treated for such in an emergency department. Social difficulties included having problems with friends and regretting sexual intercourse. In addition, multiple ROA users tended to be involved in physical fights, have problems with the police, and have caused damage to property; however, for these outcomes, only trends were found (*p* < 0.10). Our results show that, already at this young age, men who smoke illicit drugs and, in particular, those who use multiple ROAs experience important adverse outcomes. Furthermore, it appears that, with respect to the number of reported harms, the use of more than one ROA is the most problematic method of use among young Swiss men. This finding also supports our suspicion that those using multiple ROAs are, in fact, users who have more problematic consumption patterns or an elevated risk of developing them.

We also note that, despite experiencing important mental health issues and adverse health and social consequences, only a few of our subjects who reported using multiple ROAs (3.7%) also reported having been treated at a specialised centre for problems of dependence. Of those who consumed illicit drugs by smoking only, none had ever had contact with such a treatment centre. Given that we did not collect detailed information on treatment seeking, we cannot provide reasons for the low percentage of subjects with prior contact with a treatment centre. It is possible that the young men in the present study still did not perceive these adverse outcomes as disabling or acknowledge the disadvantages associated with them. For instance, in a previous study that compared young drug users who were unknown to treatment agencies against those in treatment, perceiving one’s own state of health as good and not feeling the need for treatment were the reasons most frequently given for not seeking treatment [38]. Furthermore, the ORs that we observed suggest that the effect sizes of these associations were mostly small (ORs of 1.5) and few were medium (ORs of 2.5) [33]. The strength of these associations may imply that the young men in our sample were at an early stage of their pathway to drug use. Nevertheless, without adequate treatment, impairments related to drug addiction and disadvantages associated with adverse outcomes may increase over time, also making it more difficult for young men to cope with the many challenges that arise during young adulthood. Considering our participants’ youth, together with the observations that around half used their drugs either through smoking or, more importantly, through multiple routes and that they currently were not yet seeking treatment, our findings are alarming. Efforts should be intensified to provide strategies and counselling that help to prevent and reduce the use of illicit drugs, both by smoking and multiple ROAs. In this context, it is important to provide strategies that help drug users to identify signs of potentially-problematic drug use and to recognise the need for assistance before they develop actually problematic patterns of use.

### 4.3. Limitations

The following limitations of our study must be considered. First, women were not included in our study, even though evidence exists that women and men may differ in their preference for ROAs [3,12]. Therefore, the prevalence estimates reported in the present study cannot be generalised to females. Second, since the data on ROAs and adverse outcomes were collected at the same time point, we cannot draw any causal inferences. Third, we collected data from a sample drawn from the general population. In such surveys, it is difficult to generate unbiased prevalence estimates associated with the use of illicit drugs. This is especially true for more problematic forms of drug use, such as injecting. This is due to the low social acceptance of such behaviours, but also to difficulties capturing subjects with particular characteristics who more often engage in such behaviours—for example, those who are homeless or in prison [1,6,16,35]). As a consequence, the true prevalence of ROAs might have been slightly underestimated in our study. Fourth, the number of men who used illicit drugs only by smoking was small. As such, for this particular category of use, some socio-demographic and adverse outcomes cells only captured a few cases. It is, therefore, possible that our findings might have underestimated the strength of the relationship between smoking illicit drugs and particular socio-demographic variables and adverse outcomes. These associations must be examined further in future studies using larger samples and/or longitudinal data, or applying other methodological approaches, such as case–control studies. Fifth, we did not correct for the number of drugs used because, for this variable, we did not have enough cases for all the categories related to the ROAs of interest to conduct reliable analyses (e.g., only three men used more than one drug by smoking). However, as mentioned previously, the use of multiple ROAs and multiple drugs seem to be strongly related and must be addressed more thoroughly in larger samples. Finally, we focused on socio-demographic characteristics and adverse outcomes specific to the ROAs, without taking different types of drugs into account. Given that some illicit drugs were used more prevalently than others and that they were primarily used by specific ROAs, it is possible that the effect of these drugs might have influenced the strength of the associations observed. Although we believe that the current study provides an overview of the ROAs used among young men and of potentially related socio-demographic characteristics and outcomes, further studies using larger samples and/or longitudinal data remain necessary to allow a deeper investigation into the relationship between the method of use and the number and type of drug used.

## 5. Conclusions

In conclusion, among young Swiss men, the most commonly used routes of drug administration appear to be oral followed by nasal use and smoking. In terms of ROA, the most problematic pattern of drug use among young adults appears to be their administration using more than one route. This method of use was reported by roughly half of our subjects and was associated with several adverse outcomes, including mental health problems (i.e., AUD and suicidal ideations), as well as adverse physical health and social consequences. The second most problematic ROA was smoking, which also was linked to suicidal ideations and adverse health consequences. Strategies to prevent and reduce the use of illicit drugs, both by smoking and multiple ROAs, are crucial to avoid adverse outcomes at this young age and reduce the risk of additional negative effects during later phases of life. Considering that men start to experiment more intensively with illicit drugs beyond cannabis during young adulthood, such strategies may be more successful when administered before individuals enter this critical stage of life.

## Figures and Tables

**Table 1:** Prevalence rates for ROAs among users of any of the 18 illicit drugs and for each of the 18 illicit drugs separately.

			Oral Use		Nasal Use		Smoking		Injecting		Other ROAs	
	*n*	%	*n*	%	*n*	%	*n*	%	*n*	%	*n*	%
Any drug use	754 *		541	71.8	446	59.2	167	22.1	8	1.1	13	1.7
Illicit substances												
ecstasy (MDMA)	435	57.7	411	94.5	53	12.2	5	1.1	0	0.0	2	0.5
cocaine, crack, freebase	360	47.7	28	7.8	322	89.4	23	6.4	3	0.8	0	0.0
amphetamine/speed, amphetamine sulphate (e.g., dexedrine, benzedrine)	228	30.2	49	21.5	195	85.5	3	1.3	0	0.0	0	0.0
natural hallucinogens (magic mushrooms, psilocybin, peyote, mescaline	176	23.3	167	94.9	1	0.6	5	2.8	1	0.6	2	1.1
poppers (amyl nitrite, butyl nitrite)	149	19.8	3	2.0	101	67.8	42	28.2	0	0.0	1	0.7
other synthetic hallucinogens (LSD, PCP, angel dust, 2-CB, 2-CI)	143	19.0	130	90.9	9	6.3	3	2.1	0	0.0	0	0.0
solvent sniffing (e.g., glue, solvent and gases such as benzine, ether, toluol, trichloroethylene, nitrous oxide)	71	9.4	19	26.8	10	14.1	37	52.1	0	0.0	3	4.2
ketamine (special K), DXM (Bexin)	59	7.8	3	5.1	51	86.4	2	3.4	0	0.0	0	0.0
salvia divinorum	33	4.4	7	21.2	0	0.0	24	72.7	0	0.0	1	3.0
ayahuasca/DMT, psychedelic rainforest plants	33	4.4	9	27.3		0.0	23	69.7		0.0	1	3.0
heroin, morphine, opium	26	3.4	8	30.8	5	19.2	13	50.0	6	23.1	1	3.8
spices or similar substances to smoke which can contain synthetic cannabinoids	24	3.2	8	33.3	0	0.0	13	54.2	0	0.0	0	0.0
methamphetamine (Thai pills, crystal meth (ice))	23	3.1	4	17.4	5	21.7	11	47.8	1	4.3	0	0.0
GHB/GBL/BDO	21	2.8	17	81.0	0	0.0	1	4.8	0	0.0	1	4.8
bath salts, research chemicals, other legal highs (e.g., MDPV, mephedrone, butylone, and methedrone)	10	1.3	3	30.0	7	70.0	0	0.0	0	0.0	0	0.0
khat	6	0.8	4	66.7	1	16.7	0	0.0	0	0.0	0	0.0
methadone	6	0.8	5	83.3	0	0.0	0	0.0	0	0.0	0	0.0
ibogaine	4	0.5	0	0.0	0	0.0	0	0.0	0	0.0	1	25.0

* Total number of participants (*n*) only includes participants who consumed any of the illicit drugs of interest. ROAs: routes of administration; MDMA: 3,4-methylenedioxy-N-methylamphetamine; LSD: lysergide; PCP: phencyclidine; 2-CB: 4-bromo-2,5-dimethoxyphenethylamine; 2-CI: 2,5-dimethoxy-4-iodophenethylamine; DXM: dextromethorphan; DMT: dimethyltryptamine; GHB: gamma-hydroxybutyric acid; GBL: gamma-butyrolactone; BDO: 1,4 butanediol; MDPV: methylendioxypyrovaleron; Note: Multiple responses are possible; therefore, the sum per line can exceed 100%. This sum can be also less than 100% if participants did not specify the ROA that they used, e.g., for ibogaine.

**Table 2:** Prevalence rates for the use of single and multiple ROAs among users of any of the 18 illicit drugs.

	Prevalence of ROAs among Illicit Drug Users (*n* = 754 *)	
	*n*	%
oral use only	202	26.8
nasal use only	122	16.2
smoking only	43	5.7
injecting only	3	0.4
use of other ROAs only	3	0.4
use of multiple ROAs	360	47.7
no ROA specified	21	2.8

* Total number of participants (*n*) only includes participants who consumed any of the illicit drugs of interest. ROAs: routes of administration.

**Table 3:** Socio-demographic characteristics according to routes of administration among users of any of the 18 illicit drugs.

	All Participants			Route of Administration					
	Total ^a^	*n*	%	Oral Use Only	Nasal Use Only	Smoking Only	Multiple ROAs	χ^2^	*p*
Total	711			199	120	42	350		
Age	711								
<25		228	32.1	37.2	26.7	31.0	31.1	4.16	0.245
≥25		483	67.9	62.8	73.3	69.0	68.9		
Linguistic region	711								
French		443	62.3	61.8	66.7	73.8	59.7	4.36	0.225
German		268	37.7	38.2	33.3	26.2	40.3		
Education level	711								
High school/university		404	56.8	64.8	52.5	61.9	53.1	8.48	0.037
Primary school/secondary vocational school		307	43.2	35.2	47.5	38.1	46.9		
Degree of financial autonomy	711								
Financial autonomy		335	47.1	41.2	58.3	59.5	45.1	17.44	0.008
Partial financial dependency		272	38.3	41.2	31.7	19.0	41.1		
Financial dependency		104	14.6	17.6	10.0	21.4	13.7		
Civil status	711								
Single		636	89.5	91.5	90.0	78.6	89.4	5.52 ^b^	0.132
In relationship		75	10.5	8.5	10.0	21.4	10.6		

^a^ Total number of participants who consumed any of the illicit drugs of interest (*n*) recorded for this variable. ^b^ Analyses were performed with Fisher’s exact test.

**Table 4:** Multinomial logistic regression analyses with routes of administration as outcome. The analyses were performed for users of any of the 18 illicit drugs.

	Nasal Use Only ^a^		Smoking Only ^a^		Use of Multiple ROAs ^a^	
	Crude OR [CI]	AOR ^b^ [CI]	Crude OR [CI]	AOR ^b^ [CI]	Crude OR [CI]	AOR ^b^ [CI]
Age						
<25	1.00	1.00	1.00	1.00	1.00	1.00
≥25	1.63 [0.99–2.67] †	1.32 [0.78–2.23]	1.32 [0.65–2.70]	1.02 [0.48–2.20]	1.31 [0.91–1.89]	1.23 [0.84–1.81]
Linguistic region						
French	1.00	1.00	1.00	1.00	1.00	1.00
German	0.81 [0.50–1.30]	0.82 [0.50–1.34]	0.57 [0.27–1.21]	0.6 [0.28–1.31]	1.09 [0.76–1.56]	1.16 [0.80–1.69]
Education level						
High school/university	1.00	1.00	1.00	1.00	1.00	1.00
Primary school /secondary vocational school	1.67 [1.05–2.65] *	1.25 [0.75–2.09]	1.13 [0.57–2.26]	0.77 [0.36–1.64]	1.62 [1.14–2.33] **	1.63 [1.09–2.44] *
Degree of financial autonomy						
Financial autonomy	1.00	1.00	1.00	1.00	1.00	1.00
Partial financial dependency	0.54 [0.33–0.89] *	0.62 [0.36–1.07]†	0.32 [0.14–0.75] **	0.31 [0.12–0.77] *	0.91 [0.62–1.33]	1.19 [0.78–1.83]
Financial dependency	0.4 [0.19–0.83] *	0.47 [0.22–1.01]†	0.84 [0.36–1.99]	0.8 [0.31–2.06]	0.71 [0.43–1.19]	0.96 [0.55–1.66]
Civil status						
In relationship	1.00	1.00	1.00	1.00	1.00	1.00
Single	0.84 [0.39–1.83]	1.04 [0.47–2.30]	0.34 [0.14–0.83] *	0.42 [0.17–1.05] †	0.79 [0.43–1.44]	0.83 [0.45–1.54]

ROAs: routes of administration. † *p* < 0.10, * *p* < 0.05, ** *p* < 0.01; OR: odds ratio; CI: 95% confidence interval. ^a^ Reference category: oral use only. ^b^ Adjusted for all socio-demographic variables. Note: ORs of 1.5 or 0.67 correspond to a small effect size, ORs of 2.5 or 0.4 correspond to a medium effect, and ORs of 4 or 0.25 correspond to a large effect [33].

**Table 5:** Adverse outcomes according to routes of administration among users of any of the 18 illicit drugs.

	All Participants			Route of Administration					
	Total ^a^	*n*	%	Oral Use Only	Nasal Use Only	Smoking Only	Multiple ROAs	χ^2^	*p*
Total	711			199	120	42	350		
Mental health outcomes									
AUD	710								
yes		436	61.4	55.3	60.0	50.0	66.8	9.78	0.020
Suicidal ideations	708								
yes		234	33.1	27.6	30.0	39.0	36.5	5.66	0.129
Social consequences									
physical fights	711								
at least once		178	25.0	20.6	24.2	16.7	28.9	6.42	0.093
problems with parents/family	711								
at least once		167	23.5	20.1	23.3	23.8	25.4	2.01	0.571
problems with friends	711								
at least once		234	32.9	27.6	25.8	33.3	38.3	9.81	0.020
poor performance at school/work	710								
at least once		328	46.2	42.7	50.0	50.0	46.4	1.92	0.589
theft	711								
at least once		108	15.2	13.1	11.7	14.3	17.7	3.61	0.307
problems with the police	711								
at least once		122	17.2	14.1	10.0	9.5	22.3	13.85	0.003
regretted sexual intercourse	711								
at least once		214	30.1	26.1	29.2	23.8	33.4	4.17	0.243
damage to property	711								
at least once		193	27.1	24.6	20.0	19.0	32.0	9.30	0.026
Health consequences									
accident/injury	711								
at least once		399	56.1	48.7	56.7	61.9	59.4	6.54	0.088
admittance to an ED	711								
at least once		142	20.0	15.1	20.8	19.0	22.6	4.54	0.208
need for medical treatment	710								
at least once		390	54.9	50.5	52.5	69.0	56.6	5.61	0.132
overnight stay in a hospital	710								
at least once		60	8.5	6.0	5.8	16.7	9.7	6.61 ^b^	0.078
outpatient surgery	711								
at least once		64	9.0	7.5	8.3	9.5	10.0	1.02 ^b^	0.785
treatment of an accident/injury in an ED	711								
at least once		149	21.0	14.1	16.7	31.0	25.1	13.26	0.004
treatment at a specialised centre for problems of dependence of toxic substances	711								
at least once		20	2.8	2.5	1.7	0.0	3.7	1.87 ^b^	0.560

AUD: Alcohol use disorder; ED: emergency department. ^a^ Total number of participants who consumed any of the illicit drugs of interest (*n*) recorded for this variable; *n* varies slightly between variables due to missing data. ^b^ Analyses were performed with Fisher’s exact test.

**Table 6:** Logistic regression analyses with mental health outcomes and social and health consequences as outcomes. The analyses were performed for users of any of the 18 illicit drugs.

Mental health outcomes				
	AUD		Suicidal Ideations	
	Crude OR [CI]	AOR ^a^ [CI]	Crude OR [CI]	AOR ^a^ [CI]
ROAs				
oral use	1.00	1.00	1.00	1.00
nasal use	1.21 [0.77–1.92]	1.29 [0.81–2.06]	1.12 [0.68–1.85]	1.21 [0.73–2.03]
smoking	0.81 [0.42–1.58]	0.94 [0.47–1.85]	1.68 [0.83–3.37]	2.13 [1.03–4.40] *
MuROA	1.63 [1.14–2.32] **	1.66 [1.15–2.38] **	1.5 [1.03–2.20] *	1.49 [1.01–2.20] *
Social consequences				
	physical fights		problems with parents/family	
	Crude OR [CI]	AOR ^a^ [CI]	Crude OR [CI]	AOR ^a^ [CI]
ROAs				
oral use	1.00	1.00	1.00	1.00
nasal use	1.23 [0.71–2.11]	1.04 [0.60–1.81]	1.21 [0.70–2.09]	1.29 [0.74–2.27]
smoking	0.77 [0.32–1.86]	0.72 [0.29–1.77]	1.24 [0.56–2.74]	1.35 [0.60–3.06]
MuROA	1.56 [1.03–2.37] *	1.45 [0.95–2.22] †	1.36 [0.89–2.07]	1.37 [0.89–2.12]
	problems with friends		poor performance at school/work	
	Crude OR [CI]	AOR ^a^ [CI]	Crude OR [CI]	AOR ^a^ [CI]
ROAs				
oral use	1.00	1.00	1.00	1.00
nasal use	0.91 [0.55–1.52]	0.93 [0.55–1.57]	1.34 [0.85–2.11]	1.45 [0.91–2.30]
smoking	1.31 [0.64–2.67]	1.53 [0.73–3.17]	1.34 [0.69–2.61]	1.44 [0.73–2.83]
MuROA	1.62 [1.11–2.37] *	1.62 [1.10–2.39] *	1.16 [0.82–1.65]	1.17 [0.82–1.67]
	theft		problems with the police	
	Crude OR [CI]	AOR ^a^ [CI]	Crude OR [CI]	AOR ^a^ [CI]
ROAs				
oral use	1.00	1.00	1.00	1.00
nasal use	0.88 [0.44–1.76]	0.87 [0.43–1.75]	0.68 [0.33–1.39]	0.59 [0.29–1.23]
smoking	1.11 [0.43–2.89]	1.11 [0.42–2.93]	0.64 [0.21–1.94]	0.61 [0.20–1.87]
MuROA	1.43 [0.87–2.35]	1.43 [0.87–2.36]	1.75 [1.09–2.81] *	1.61 [0.99–2.60] †
	regretted sexual intercourse		damage to property	
	Crude OR [CI]	AOR ^a^ [CI]	Crude OR [CI]	AOR ^a^ [CI]
ROAs				
oral use	1.00	1.00	1.00	1.00
nasal use	1.16 [0.70–1.93]	1.18 [0.70–1.97]	0.77 [0.44–1.33]	0.71 [0.40–1.25]
smoking	0.88 [0.41–1.92]	1.00 [0.45–2.21]	0.72 [0.31–1.66]	0.67 [0.28–1.57]
MuROA	1.42 [0.96–2.09] †	1.49 [1.00–2.21] *	1.44 [0.97–2.13] †	1.41 [0.94–2.11] †
Health consequences				
	accident/injury		admittance to an ED	
	Crude OR [CI]	AOR ^a^ [CI]	Crude OR [CI]	AOR ^a^ [CI]
ROAs				
oral use	1.00	1.00	1.00	1.00
nasal use	1.38 [0.87–2.17]	1.24 [0.78–1.97]	1.48 [0.82–2.67]	1.34 [0.73–2.44]
smoking	1.71 [0.86–3.38]	1.63 [0.81–3.27]	1.33 [0.56–3.14]	1.22 [0.50–2.95]
MuROA	1.54 [1.08–2.19] *	1.44 [1.01–2.06] *	1.64 [1.03–2.61] *	1.53 [0.95–2.45] †
	need for medical treatment		overnight stay in a hospital	
	Crude OR [CI]	AOR ^a^ [CI]	Crude OR [CI]	AOR ^a^ [CI]
ROAs				
oral use	1.00	1.00	1.00	1.00
nasal use	1.08 [0.69–1.71]	1.18 [0.74–1.89]	0.97 [0.37–2.52]	0.79 [0.30–2.11]
smoking	2.19 [1.07–4.45] *	2.39 [1.15–4.97] *	3.12 [1.15–8.47] *	2.67 [0.95–7.50] †
MuROA	1.28 [0.90–1.81]	1.34 [0.94–1.93]	1.68 [0.85–3.33]	1.47 [0.73–2.94]
	outpatient surgery		treatment of accident/injury in an ED	
	Crude OR [CI]	AOR ^a^ [CI]	Crude OR [CI]	AOR ^a^ [CI]
ROAs				
oral use	1.00	1.00	1.00	1.00
nasal use	1.12 [0.48–2.57]	0.91 [0.39–2.12]	1.22 [0.65–2.28]	1.11 [0.59–2.09]
smoking	1.29 [0.41–4.11]	1.22 [0.37–3.97]	2.74 [1.27–5.89] *	2.73 [1.25–5.96] *
MuROA	1.36 [0.72–2.56]	1.20 [0.63–2.28]	2.05 [1.29–3.27] **	1.98 [1.23–3.17] **

ROAs: routes of administration; MuROA: use of multiple routes of administration; ED: emergency department. † *p* < 0.10, * *p* < 0.05, ** *p* < 0.01; OR: odds ratio; CI: 95% confidence interval. ^a^ Adjusted for all socio-demographic variables. Note: ORs of 1.5 or 0.67 correspond to a small effect size, ORs of 2.5 or 0.4 correspond to a medium effect, and ORs of 4 or 0.25 correspond to a large effect [33].

## Data Availability

The data and materials from the CSURF study are available for research collaborations. More information can be found at the CSURF website: www.c-surf.ch (accessed on 30 April 2021).
